# Free‐breathing simultaneous *T*_1_, *T*_2_, and *T*_2_^∗^ quantification in the myocardium

**DOI:** 10.1002/mrm.28753

**Published:** 2021-03-29

**Authors:** Ingo Hermann, Peter Kellman, Omer B. Demirel, Mehmet Akçakaya, Lothar R. Schad, Sebastian Weingärtner

**Affiliations:** ^1^ Department of Imaging Physics Magnetic Resonance Systems Lab Delft University of Technology Delft The Netherlands; ^2^ Computer Assisted Clinical Medicine Medical Faculty Mannheim Heidelberg University Mannheim Germany; ^3^ National Heart, Lung, and Blood Institute National Institutes of Health, DHHS Bethesda MD USA; ^4^ Department of Electrical and Computer Engineering and Center for Magnetic Resonance Research University of Minnesota Minnesota MN USA

**Keywords:** cardiac quantitative imaging, free‐breathing, $T_{1}$ mapping, $T_{2}$ mapping, $T_{2}^{*}$ mapping

## Abstract

**Purpose:**

To implement a free‐breathing sequence for simultaneous quantification of $T_{1}$, $T_{2}$, and $T_{2}^{*}$ for comprehensive tissue characterization of the myocardium in a single scan using a multi‐gradient‐echo readout with saturation and $T_{2}$ preparation pulses.

**Methods:**

In the proposed Saturation And $T_{2}$‐prepared Relaxometry with Navigator‐gating (SATURN) technique, a series of multi‐gradient‐echo (GRE) images with different magnetization preparations was acquired during free breathing. A total of 35 images were acquired in 26.5 ± 14.9 seconds using multiple saturation times and $T_{2}$ preparation durations and with imaging at 5 echo times. Bloch simulations and phantom experiments were used to validate a 5‐parameter fit model for accurate relaxometry. Free‐breathing simultaneous $T_{1}$, $T_{2}$, and $T_{2}^{*}$ measurements were performed in 10 healthy volunteers and 2 patients using SATURN at 3T and quantitatively compared to conventional single‐parameter methods such as SASHA for $T_{1}$, $T_{2}$‐prepared bSSFP, and multi‐GRE for $T_{2}^{*}$.

**Results:**

Simulations confirmed accurate fitting with the 5‐parameter model. Phantom measurements showed good agreement with the reference methods in the relevant range for in vivo measurements. Compared to single‐parameter methods comparable accuracy was achieved. SATURN produced in vivo parameter maps that were visually comparable to single‐parameter methods. No significant difference between $T_{1}$, $T_{2}$, and $T_{2}^{*}$ times acquired with SATURN and single‐parameter methods was shown in quantitative measurements (SATURN $T_{1}=1573\pm 86\;\text{ms}$, $T_{2}=33.2\pm 3.6\;\text{ms}$, $T_{2}^{*}=25.3\pm 6.1\;\text{ms}$; conventional methods: $T_{1}=1544\pm 107\;\text{ms}$, $T_{2}=33.2\pm 3.6\;\text{ms}$, $T_{2}^{*}=23.8\pm 5.5\;\text{ms}$; P>.2)

**Conclusion:**

SATURN enables simultaneous quantification of $T_{1}$, $T_{2}$, and $T_{2}^{*}$ in the myocardium for comprehensive tissue characterization with co‐registered maps, in a single scan with good agreement to single‐parameter methods.

## 1 Introduction

Quantitative mapping in the myocardium has received major clinical interest, as markers related to myocardial relaxation time yield promising sensitivity to a broad spectrum of cardiomyopathies. $T_{1}$, $T_{2}$, and $T_{2}^{*}$ mapping are routinely used in advanced CMR centers and received increasing interest in community recommendations and consensus statements for the assessment of ischemia, fibrosis, edema, and amyloidosis or iron deposition.^1^, ^2^, ^3^, ^4^

A wide variety of mapping sequences was proposed in the last decades for noninvasively studying the myocardial tissue state.^5^, ^6^, ^7^, ^8^, ^9^ Myocardial $T_{1}$ mapping is most commonly performed based on a series of inversion or saturation recovery images and has shown promise for the assessment of ischemic and nonischemic cardiomyopathies.^1^, ^4^, ^10^, ^11^ While inversion recovery‐based methods have shown improved precision and map quality, saturation recovery methods yield more accurate $T_{1}$ maps insensitive to the heart rate, the magnetization evolution, and other confounders.^12^, ^13^, ^14^

In addition to $T_{1}$ mapping, myocardial $T_{2}$ mapping is increasingly used for the reliable assessment of myocardial edema.^15^ State of the art cardiac $T_{2}$ mapping is performed by acquiring at least 3 $T_{2}$‐prepared balanced steady‐state free precession (bSSFP) images to provide robust and reproducible $T_{2}$ maps.^15^, ^16^, ^17^, ^18^

Myocardial $T_{2}^{*}$ quantification has demonstrated high clinical value for the assessment of myocardial iron accumulation.^19^, ^20^, ^21^ According to relevant guidelines, $T_{2}^{*}$ measurements in the myocardium is most commonly performed by acquiring 8 echoes with a multi‐gradient‐echo readout and performing an exponential fit.^19^

The methods described above each require one breath‐hold per slice. Therefore, free‐breathing methods and simultaneous quantification of $T_{1}$ and $T_{2}$ were proposed to improve patient comfort and shorten measurement time.^22^, ^23^, ^24^, ^25^, ^26^, ^27^, ^28^, ^29^, ^30^ Simultaneous $T_{1}$ and $T_{2}$ mapping was obtained in a single breath‐hold by combining saturation/inversion pulses and $T_{2}$ preparation modules to improve the detection of abnormalities by inherently co‐registered parametric maps.^22^, ^31^, ^32^ This method was expanded to a navigator gated free‐breathing approach allowing the coverage of $T_{1}$ and $T_{2}$ in the entire myocardium in a single scan avoiding deviations due to incorrect breath‐holds.^23^, ^33^ Magnetic resonance fingerprinting was proposed for joint estimation of $T_{1}$ and $T_{2}$ based on undersampled non‐Cartesian readouts with varying preparations.^25^ Most recently, cardiac multitasking was introduced, as a novel method for multiparameter mapping, where contrast and physiological variations are modeled by a low‐dimensional representation, enabling a continuous acquisition of multiparametric 3D maps.^24^

However, the lack of a combined method for assessment of all 3 clinically relevant tissue characteristics ($T_{1}$, $T_{2}$, and $T_{2}^{*}$) requires multiple sequences in clinical practice, expanding the scan protocol and prolonging examination duration. Furthermore, many recently developed methods rely on implicit or explicit model‐based regularization.^34^, ^35^ This often induces quantification inaccuracies and renders the methods’ quantification susceptible to changes in the reconstruction pipeline.

In this study, we sought to provide a method for free‐breathing assessment of all clinically relevant relaxation times ‐ $T_{1}$, $T_{2}$, and $T_{2}^{*}$. A navigator gated sequence with multi‐gradient‐echo readout and saturation and $T_{2}$ preparation pulses is developed. The accuracy of the proposed technique is evaluated in phantom measurements and in vivo image quality is assessed in healthy subjects and a small cohort of patients.

## 2 METHODS

### 2.1 Sequence design

**FIGURE 1:**
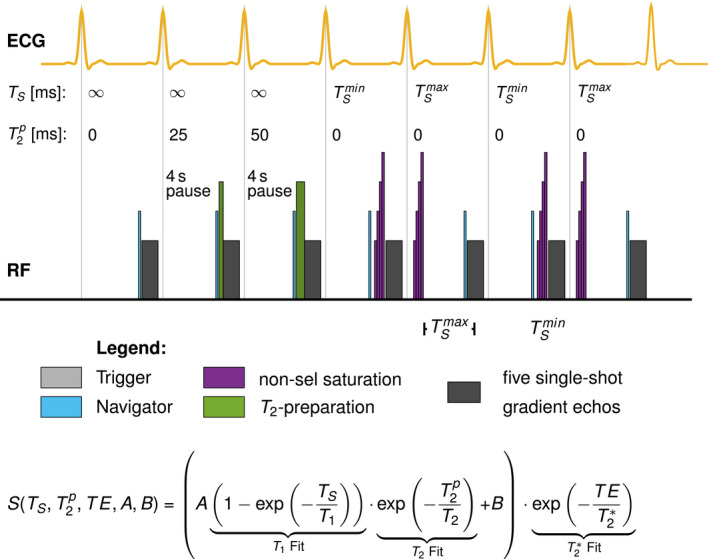
Sequence diagram for the proposed $T_{1}$, $T_{2}$, and $T_{2}^{*}$ mapping technique. Navigator pulses (light blue) are played before the readouts and the preparation pulses. Five different multi‐gradient‐echoes per imaging block are generated. The first contrast is performed without any preparation pulses to image the fully relaxed magnetization signal. Second and third contrasts are prepared with 25 and 50 ms $T_{2}$ preparation pulses comprising composite hard pulses,^37^ respectively. A non‐selective saturation recovery (WET) pulse is performed immediately ($T_{S}^{\min}$) before the readout of contrasts number 4 and 6. The same preparation pulse is played in the systole for contrasts 5 and 7, facilitating longer $T_{1}$ relaxation^38^

Figure 1 depicts the sequence diagram of the proposed Saturation And $T_{2}$ prepared Relaxometry with Navigator‐gating (SATURN) sequence. The sequence is based on a single‐shot multi‐gradient‐echo readout generating 5 echoes for each end‐diastolic imaging window. We used a prospective navigator on the diaphragm of the liver with a gating window of 4‐5 mm depending on the subject’s breathing pattern. Navigator gating is performed with the following accept‐reject scheme: The first contrast without preparation was repeated if the navigator was rejected. Saturation prepared images were also immediately re‐attempted in the next heartbeat. No navigator was played during the rest periods before the $T_{2}$ preparation. For $T_{2}$‐prepared images, $T_{2}$ preparation was only performed if the navigator was accepted. In this way, if the navigator was rejected the $T_{2}$‐prepared image could be re‐attempted immediately, without the need of additional rest‐periods. However, in this way, navigator rejections lead to an increase in effective rest periods. We used saturation and $T_{2}$ preparation pulses before the readouts to generate $T_{1}$ and $T_{2}$ contrasts. Therefore, we combined the SASHA 3‐parameter fit model with the $T_{2}$‐prepared bSSFP 3‐parameter fit model. Since we only use short echo times (TE) for the gradient‐echo readout and the noise floor for the $T_{2}^{*}$ decay is not corrected, we used a truncation model for $T_{2}^{*}$ as previously suggested.^36^ The 5‐parameter truncation fit model is given as

(1) $$\begin{array}{c} S\left(T_{S},T_{2}^{p},TE,A,B\right)=\left(A\underset{\text{SASHA Fit}}{\underbrace{\left(1‐\exp\left(‐\frac{T_{S}}{T_{1}}\right)\right)}}\cdot \underset{T_{2}\;\text{Fit}}{\underbrace{\exp\left(‐\frac{T_{2}^{p}}{T_{2}}\right)}}+B\right)\cdot \underset{T_{2}^{*}\;\text{Fit}}{\underbrace{\exp\left(‐\frac{\mathrm{TE}}{T_{2}^{*}}\right)}}. \end{array}$$

Here, the fitting parameter B is used to account for the $T_{1}$ offset. Thus, $T_{2}^{*}$ is reconstructed with a truncation model. The first contrast is performed without any preparation representing full magnetization recovery (infinite saturation time, $T_{S}$) and $T_{2}$ preparation time of $T_{2}^{p}=0$. The second block consists of 2 different $T_{2}$‐weighted contrasts using preparation durations of 25 and 50 ms, respectively, as previously recommended.^22^ Four seconds of rest period were inserted before each image without saturation preparation to allow for full magnetization recovery. Due to the rest‐periods, full magnetization recovery was assumed prior to the $T_{2}$ preparation. The third block acquires images with saturation preparation to sample the $T_{1}$ recovery curve. The fourth and sixth image is performed with a saturation pulse before the readout to mimic the effect of a very long $T_{2}$ preparation^37^ and short saturation times and, thus, $T_{S}$ and $T_{2}^{p}$ was set to $T_{S}^{\text{min}}$ and 0. Image 5 and 7 are acquired with saturation preparation with a maximum $T_{S}$ for maximum precision.^38^

The full acquisition comprises 7 different contrast preparations followed by imaging at 5 echo times, yielding a total of 35 images. Saturation pulses were performed using a composite “Water suppression Enhanced through $T_{1}$‐effects” (WET) pulse to reduce the sensitivity to $B_{1}$.^39^ The $T_{2}$ preparation module consist of a $90^{\circ }$ rectangular flip‐down pulse, a $270^{\circ }$ rectangular flip‐up pulse and composite $180^{\circ }$ MLEV refocusing pulses in between.^40^, ^41^ Centric k‐space reordering was used for increased signal‐to‐noise ratio and shorter minimum saturation times.

### 2.2 Sequence parameters

All measurements were performed on a 3T MRI scanner (Magnetom Skyra, Siemens Healthineers, Erlangen, Germany) with a 28‐channel receiver coil array. Sequence parameters are listed in Table 1.

**TABLE 1:** Sequence parameters for SATURN and the reference methods (SASHA, $T_{2}$‐prepared bSSFP, multi‐GRE)

Parameters	SATURN	SASHA	$T_{2}$ bSSFP	multi‐GRE
FOV	$384\times 288\;{\text{mm}}^{2}$			
In‐plane res.	$2\times 2\;{\text{mm}}^{2}$			
Slice thickness	8 mm			
Partial Fourier	6/8			
Readout	Multi‐GRE	bSSFP	bSSFP	multi‐GRE
Flip angle	20^∘^	45^∘^	45^∘^	20^∘^
acq. k‐Space lines	36	66	66	11
Bandwidth	1530 Hz/px	1130 Hz/px	1130 Hz/px	965 Hz/px
GRAPPA	*R* = 3 or 4	R=2		
Respiration	Free‐breathing	Breath‐hold (exhaled)		
Number of echoes	5	1	1	8
TE	1.0‐8.5 ms	1.3 ms	1.3 ms	1.6‐16.3 ms
TR	10.3 ms	2.7 ms	2.7 ms	18.1 ms
Nom. acquisition time	18.5 s	10 s	10 s	8 s

*Note*: Common parameters are depicted with blue shading. Nominal acquisition time is calculated for a heart rate of 60 bpm and a gating efficiency of 50%.

SATURN was performed using GRAPPA with acceleration factor *R* = 3. Additionally, GRAPPA with acceleration *R* = 4 was explored for the use in subjects with higher heart rates. SPIRiT^42^ with locally low rank (LLR) reconstruction was used for improved noise‐resilience at acceleration *R* = 4, as previously proposed.^43^, ^44^, ^45^

### 2.3 Simulations

Bloch simulations were used to calculate the magnetization of the proposed SATURN sequence and validate the accuracy of the quantification. All pulse sequences were simulated with the above listed sequence parameters. The magnetization was simulated with time‐steps of 0.1 ms. Imaging and preparation pulses were simulated with corresponding rotation matrices with 100% efficiency. The center of the k‐space was chosen to extract the signal magnitude. $T_{1}$ (1200‐1700 ms), $T_{2}$ (20‐70 ms), and $T_{2}^{*}$ (5‐60 ms) were varied and the magnitude was fitted with the proposed 5‐parameter fit model given in Equation (1). Four confounding factors were included in the simulations: Rest periods before the $T_{2}$ preparation pulses were varied between 1 and 10 seconds. For all other simulations, 10 seconds were used to eliminate insufficient recovery as the primary source of inaccuracy. Image noise was added to the simulations. Rician noise was generated with an SNR between 0 and 30 and a Monte Carlo size of 1000. Different heart rates were simulated between 50 and 140 bpm. Finally, imperfect $T_{2}$ preparation was simulated by reducing the flip angle of the flip‐down and flip‐up pulses.

### 2.4 Phantom experiments

Phantom measurements were performed to evaluate the accuracy and precision of the proposed SATURN sequence. Reference measurements for $T_{1}$ were performed using an inversion‐recovery spin echo sequence with $T_{I}$ = 100, 200, 500, 1000, 2000, 5000, 8000 ms, TE/TR = 12/10 000 ms, and imaging geometry as specified above. $T_{2}$ reference scans were performed with a spin echo sequence with TE = 17, 30, 50, 100, 150, 250 ms and otherwise identical imaging parameters to the inversion recovery spin‐echo (IR‐SE). GRE was performed for $T_{2}^{*}$ quantification with 12 contrasts ranging from TE = 2‐60 ms, TR = 10 000 ms and 1 k‐space line per readout with the same imaging parameters listed above. All measurements were additionally compared with single‐parameter methods for myocardial mapping (listed in Table 1): SASHA $T_{1}$ ^46^ with a minimum and maximum saturation time of 103 ms and 600 ms, $T_{2}$‐prepared bSSFP using 4 different $T_{2}$ weightings (0 ms, 25 ms, 50 ms, and ∞ ms) and a 3‐parameter fit model,^22^, ^47^ and multi‐GRE $T_{2}^{*}$ with 8 echoes ranging from 1.6 to 16.3 ms^19^ using the 2‐parameter truncation model.^36^ The cardiac cycle was simulated and set to a heart rate (HR) of 60 bpm.

### 2.5 In vivo experiments

In vivo measurements were performed in 10 healthy volunteers (23‐29 years old, 26.1 ± 1.5 years, heart rate: 67.2 ± 7.7 bpm, 3 female), 1 patient (69 years old, female, heart rate: 72 bpm) with hypertrophic cardiomyopathy (HCM), and 1 patient (66 years old, male, heart rate: 79 bpm) with suspected hypertensive heart disease (HHD) after written consent was obtained. All images were acquired in the mid‐ventricular short‐axis view using the parameters described in the previous section.

SATURN was performed with a maximum $T_{S}$ adjusted to the subject’s heart rate. Motion between images from different heartbeats was reduced by retrospective image registration. Rigid registration was performed with mutual information in the region of interest as the similarity metric. Voxel‐wise fitting was performed using the 5‐parameter model.

Regions of interest were manually drawn in the entire myocardium, with careful distancing to the epi‐ and endocardial borders. Bullseye plots were generated for the 6 mid‐ventricular segments of the American Heart Association (AHA) segment model.^48^

Standard deviation maps (SD maps) were generated by calculating all partial derivatives of the fit function as previously proposed.^49^ The covariance matrix is calculated by the inverse of the Hessian matrix. The square root of the sum of the diagonal entries of the covariance matrix is used as an approximation for the voxel‐wise SD of the individual parameters.

### 2.6 Statistics

The within‐segment mean and the within‐segment SD of the $T_{1}$, $T_{2}$, and $T_{2}^{*}$ times were averaged across all subjects. Additionally, the within‐segment means of the SD $T_{1}$, $T_{2}$, and $T_{2}^{*}$ times were calculated using the corresponding voxel‐wise SD maps. Intersubject variability was calculated as the SD of the within‐segment mean across all subjects. Pair‐wise comparison was performed using Student’s t‐tests using the Bonferroni correction for multiple comparisons along $T_{1}$, $T_{2}$, and $T_{2}^{*}$. Values of p less than 0.05 were considered significant. Significance between segments of the myocardium was tested using the ANOVA test. Relative deviations were compared by dividing the absolute difference between reference and SATURN with the reference.

## 3 RESULTS

### 3.1 Simulations

**FIGURE 2:**
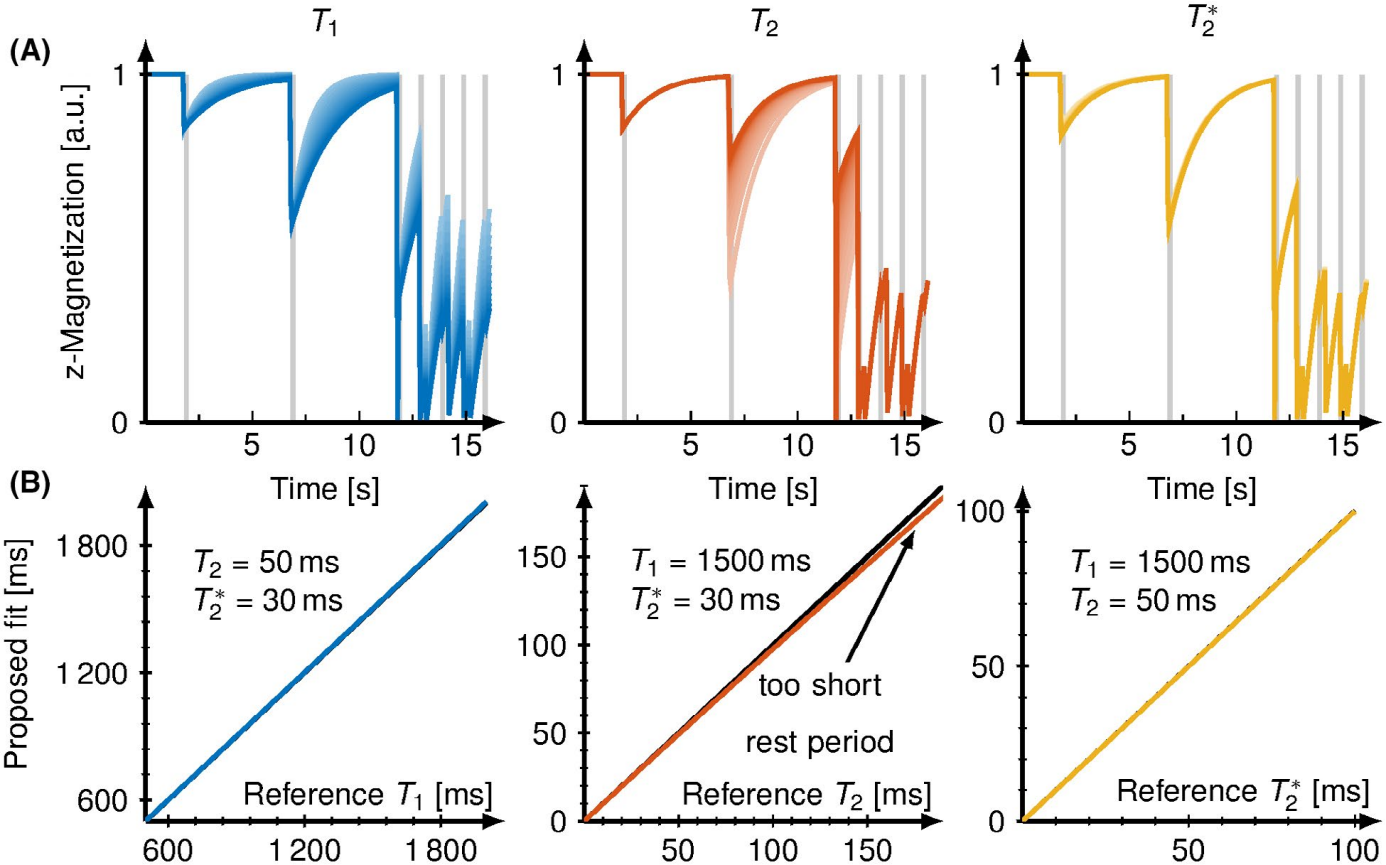
A, Simulated magnetization evaluation of the proposed sequence for varying $T_{1}$ (800‐2000 ms), $T_{2}$ (30‐100 ms), and $T_{2}^{*}$ (20‐100 ms) on the top. Increasing relaxation times are depicted by increasing brightness. B, Bottom panel shows the proposed 5‐parameter fit (blue) to the used relaxation time

Figure 2A shows the simulated longitudinal magnetization evolution of the proposed SATURN sequence with varying $T_{1}$, $T_{2}$, and $T_{2}^{*}$. Figure 2B plots the fitted relaxation times against the reference relaxation times to depict the measurement accuracy. Accurate multiparameter quantification for $T_{1}$, $T_{2}$, and $T_{2}^{*}$ across the relevant in vivo range ($T_{1}=800\text{-}2200\;\text{ms}$, $T_{2}=30\text{-}70\;\text{ms}$, $T_{2}^{*}=10\text{-}60\;\text{ms}$) was achieved in simulations. One source of deviation for $T_{2}$ was incomplete recovery during the rest‐periods leading to very slight deviations in $T_{2}$ (0.02% for 50 ms, <5% deviation for 100 ms) as shown in Supporting Information Figure S1. $T_{2}^{*}$ quantification was found to be more susceptible to higher noise levels than $T_{1}$ and $T_{2}$. $T_{1}$, $T_{2}$, and $T_{2}^{*}$ accuracy were independent of the heart rate. $T_{2}$ accuracy was additionally compromised by an imperfect $T_{2}$ preparation efficiency resulting in a strong underestimation, especially for longer $T_{2}$ times.

### 3.2 Phantom

**FIGURE 3:**
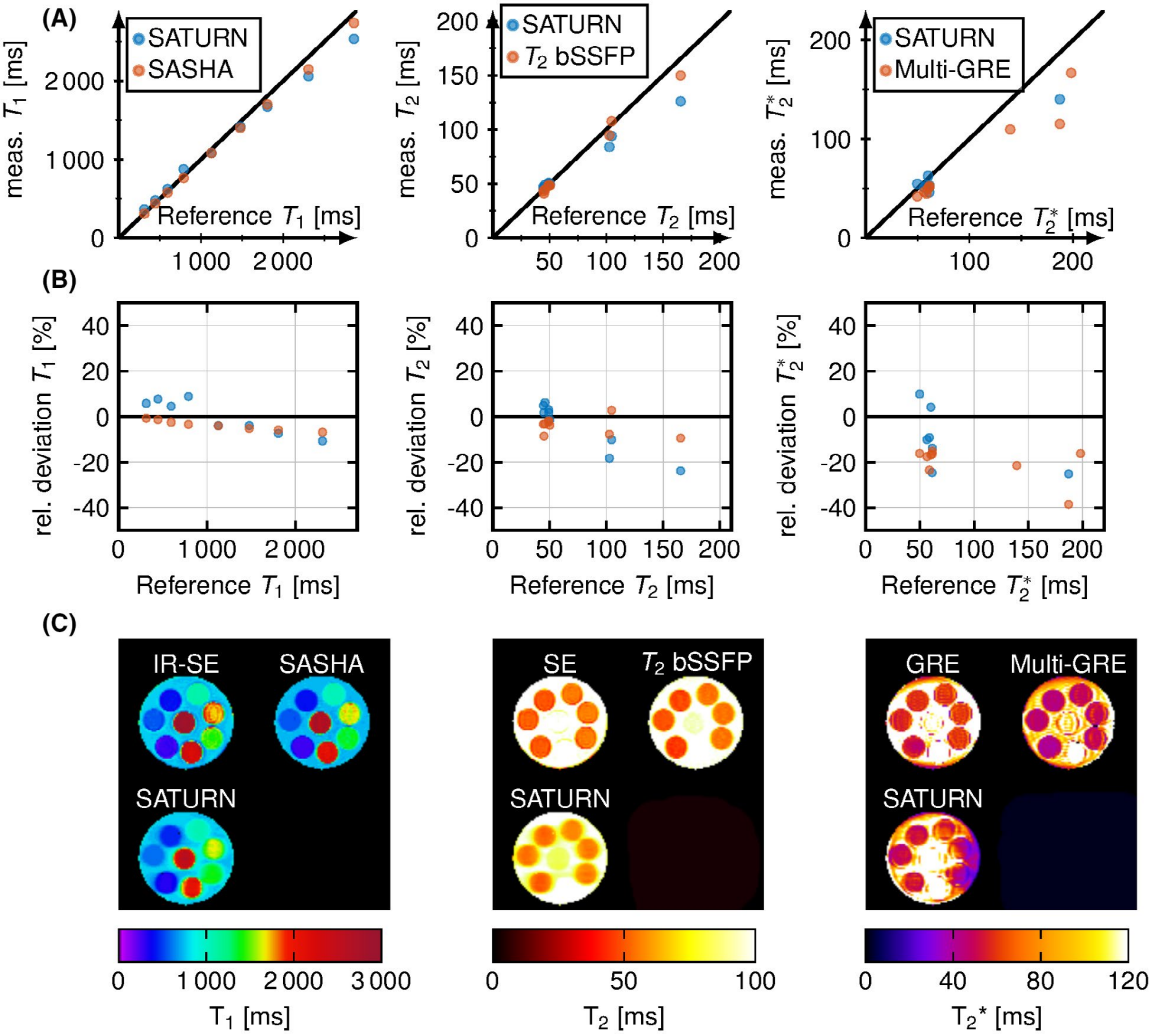
A, $T_{1}$, $T_{2}$, and $T_{2}^{*}$ acquired with SATURN (blue) and the single‐parameter methods (red) in a phantom plotted against the reference values. For $T_{2}^{*}$, 2 tubes with high relaxation times are outside of the depicted range. B, Relative difference between the reference method and SATURN and the single‐parameter models for the different relaxation times. C, Representative $T_{1}$, $T_{2}$, and $T_{2}^{*}$ maps for SATURN and the reference

Phantom measurements (Figure 3A) showed good agreement with reference methods. Deviations of less than 7.7% for relaxation times across the relevant in vivo range were observed. In Figure 3B, the relative difference of the measured relaxation times to the reference is shown as well as exemplary maps are shown for SATURN and the reference are shown below (Figure 3C). SATURN $T_{1}$ times compared with the inversion recovery spin‐echo, yielding accuracy comparable to SASHA. $T_{2}$ times were accurate in the relevant range (5.2% deviation) and decreased when exceeding 100 ms with relative deviations of up to 20%. For $T_{2}^{*}$ of less than 100 ms $T_{2}^{*}$ accuracy (7.7% deviation) was slightly higher compared with the conventional single‐parameter method, where a decrease of up to 11 ms was measured compared with the reference GRE. SATURN overestimates long $T_{2}^{*}$ times compared with the GRE and multi‐GRE.^50^ All representative relaxation times per tube are displayed in Supporting Information Table S1.

### 3.3 In vivo

**FIGURE 4:**
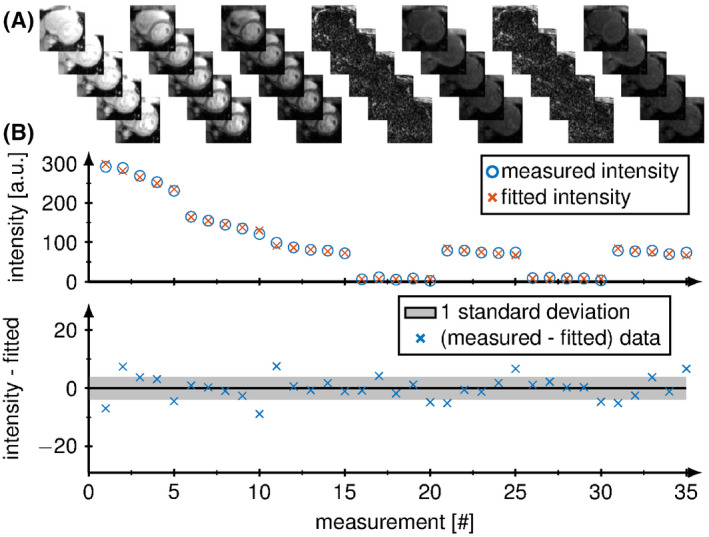
A, Magnitude images from the septum are plotted across the 35 measurements. B, Image intensities of the acquired (blue) and fitted (orange) signal model and the fit residual of a voxel in the septal myocardium are shown below where the gray area marks deviations of less than 1 standard deviation

**FIGURE 5:**
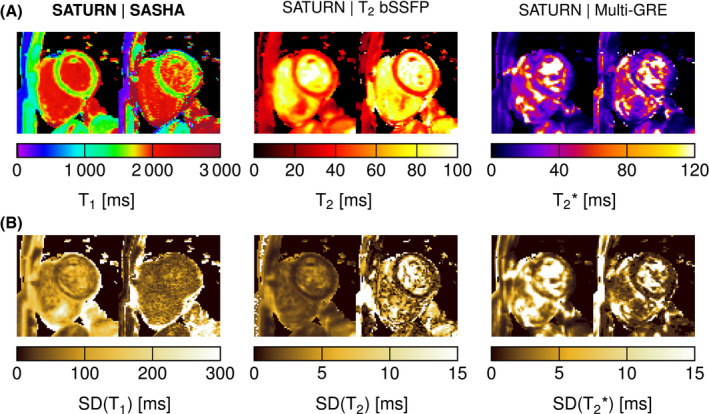
A, In vivo $T_{1}$, $T_{2}$, and $T_{2}^{*}$ maps acquired with the proposed SATURN sequence (left) and single‐parameter reference methods (right) for 1 healthy subject. Visually homogeneous mapping is achieved throughout the myocardium for $T_{1}$ and $T_{2}$, minor artifacts appear in $T_{2}^{*}$ maps. Image quality appears visually comparable to the reference methods. B, Below the standard deviation (SD) maps are shown for the 3 relaxation times and the same subject for SATURN and the reference methods

The average acquisition time for SATURN in the 10 healthy subjects was 26.5±14.9 seconds, which corresponds to an average gating efficiency of 54%±30%. The minimal $T_{S}^{\text{min}}$ was 7 ms for every subject and the maximal $T_{S}^{\max}$ was 601±65 ms. An example of magnitude data acquired with SATURN in 1 healthy subject is shown in Figure 4A. Signal intensities from the septum are plotted across 35 measurements along with the fitted signal model (Figure 4B). Visual image quality is high for $T_{1}$ and $T_{2}$. Artifacts are observed in $T_{2}^{*}$ maps (Figure 5A). SD maps depict the homogeneous mapping precision throughout the myocardium (Figure 5B).

Examples of quantitative parameter maps acquired with SATURN compared with the single‐parameter reference methods are shown in Figure 5 for 1 healthy subjects (2 more subjects are shown in Supporting Information Figure S2). Visual image quality is comparable with the single‐parameter scans for $T_{1}$ and $T_{2}$. However, some blurring is observed in the SATURN maps. $T_{1}$ and $T_{2}$ maps depict a homogeneous myocardium clear of artifacts. $T_{2}^{*}$ maps acquired with SATURN appear visually smoother than the reference.

**FIGURE 6:**
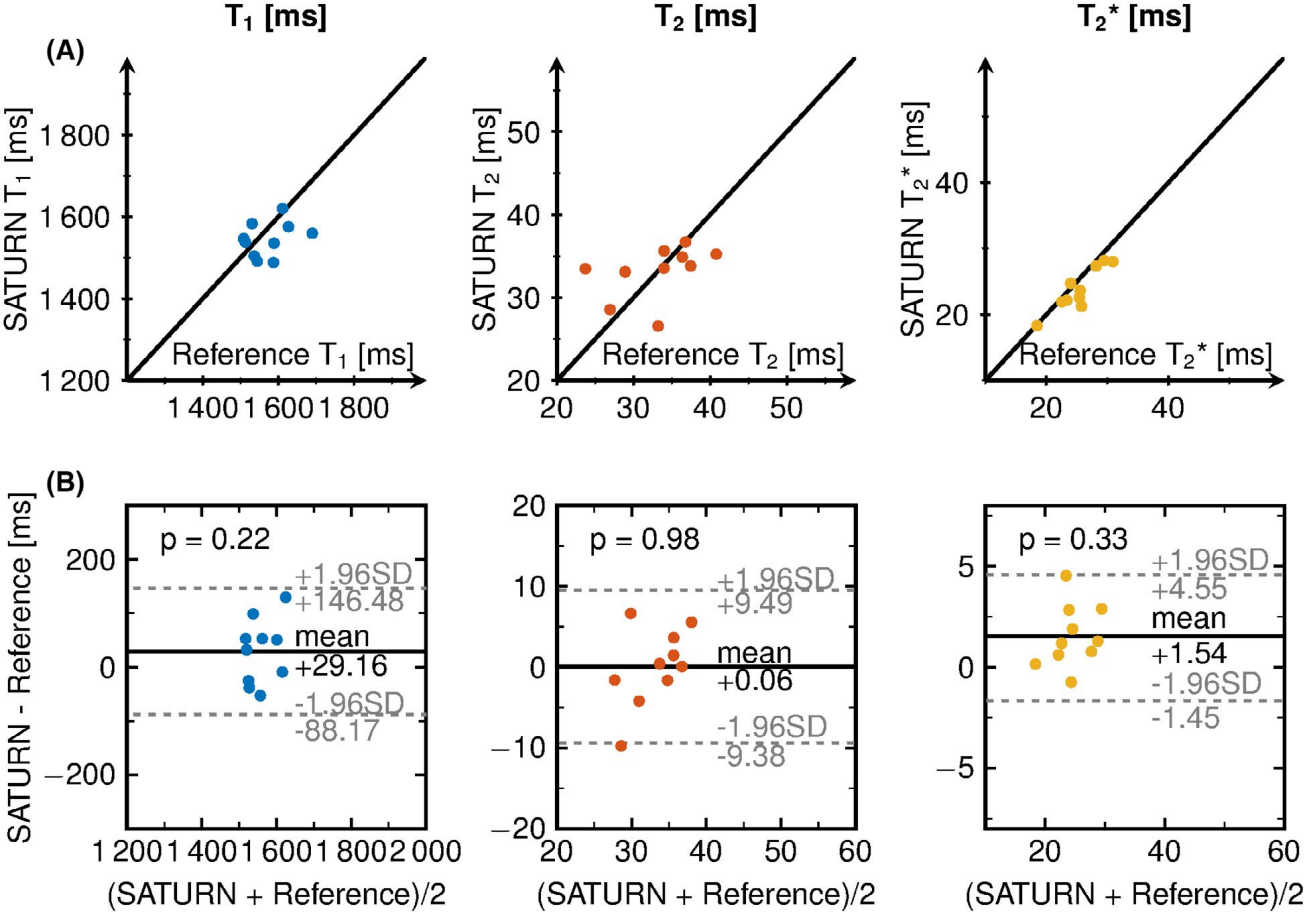
A, SATURN over the reference $T_{1}$, $T_{2}$, and $T_{2}^{*}$ times for each healthy subject. The black line shows the bisector. B, Bland‐Altman plot showing the difference between SATURN and the reference over the mean of both. The legend shows the p‐value of the Student’s t‐test

Figure 6 shows the in vivo mean $T_{1}$, $T_{2}$, and $T_{2}^{*}$ times for SATURN over the conventional methods for all healthy subjects. Below the Bland‐Altman plot is depicted. A bias of +29.16 ms was measured for $T_{1}$ and a bias of +1.54 ms was measured for $T_{2}^{*}$. $T_{2}$ times yielded negligible bias compared with $T_{1}$ and $T_{2}^{*}$ but limits of agreement of ±9.4ms. All representative relaxation times per subject are displayed in Supporting Information Table S2.

Supporting Information Figure S3 shows the difference between SATURN acquired with GRAPPA with acceleration factor R=3, R=4, and R=4 using SPIRiT + LLR regularization. $T_{2}$ map quality shows only minor differences between R=3 or R=4 with deviations of less than 2%. However, $T_{1}$ map quality is improved with 36.2% lower within‐segment SDs for R=3 compared with R=4. Precision is regained by using regularization (SPIRiT + LLR) and image quality is visually improved (only 5.4% lower within‐segment SDs). SATURN $T_{1}$ maps appear smoother and more homogeneous when using R=3 with smaller variations within the myocardium. Additional artifacts appear in $T_{2}^{*}$ maps using R=4, which are largely alleviated using regularization.

**FIGURE 7:**
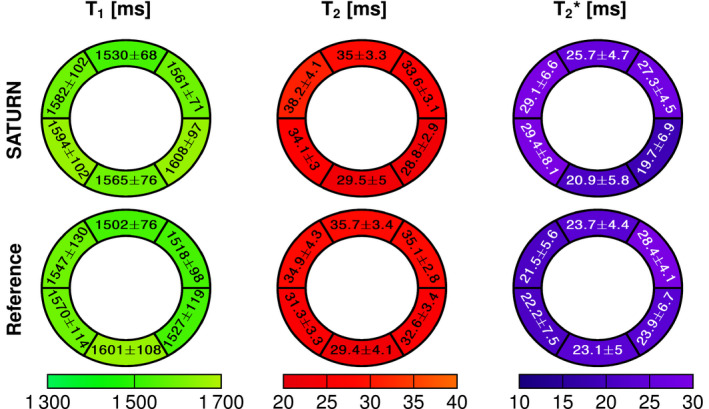
Bullseye plot of $T_{1}$, $T_{2}$, and $T_{2}^{*}$ relaxation times acquired with SATURN and the single‐parameter reference. The bullseye values are reported as the within‐segment mean ± the within‐segment standard deviation average across all healthy subjects. Small differences between SATURN and the reference was observed for $T_{1}$ and $T_{2}$. $T_{2}^{*}$ obtained with SATURN was 5.9% increased compared with the reference

Figure 7 represents the AHA 6 segment bullseye plots showing the mean quantitative measures across all healthy for the $T_{1}$, $T_{2}$, and $T_{2}^{*}$ and the corresponding within‐segment SD. The relaxation times in the healthy myocardium measured with SATURN averaged over all 6 AHA segments were $T_{1}=1573\pm 86\;\text{ms}$, $T_{2}=33.2\pm 3.6\;\text{ms}$, comparable to the conventional methods ($T_{1}=1544\pm 107\;\text{ms}$; P = .22, $T_{2}=33.2\pm 3.6\;\text{ms}$; P=.98). $T_{2}^{*}$ obtained with SATURN was 25.3±6.1ms, corresponding to a 5.9% increase compared to the conventional method (23.8±5.3ms; P=.33) with both methods suffering from artifacts. No significant differences were found between the in vivo times measured with SATURN and the conventional methods for neither $T_{1}$, $T_{2}$ or $T_{2}^{*}$.

No significant differences among segments were measured for SATURN $T_{1}$ (P=.36) but significant differences for $T_{2}$ (P=.037) and $T_{2}^{*}$ (P=.038), with the lowest $T_{2}/T_{2}^{*}$ times being observed in the mid‐inferior segment. The same trend is observed for the conventional methods. For SASHA $T_{1}$, no significant difference among the segments (P=.83) was observed, but significant differences for the single‐parameter $T_{2}$ (P=.033) and $T_{2}^{*}$ (P<.01), depicting a similar drop in the mid‐inferior segment. Intersubject variability of 57.9 ms (3.7% compared with the mean value) was observed in $T_{1}$, which is higher than for SASHA (42.3 ms (2.7%)). Intersubject variability of 3.3 ms (9.9%) for $T_{2}$ obtained with SATURN were in the range of the $T_{2}$‐prepared bSSFP with 3.2 ms (9.6%), and 3.6 ms (14.2%) for $T_{2}^{*}$ compared with the multi‐GRE 3.2 ms (13.4%) were observed.

**FIGURE 8:**
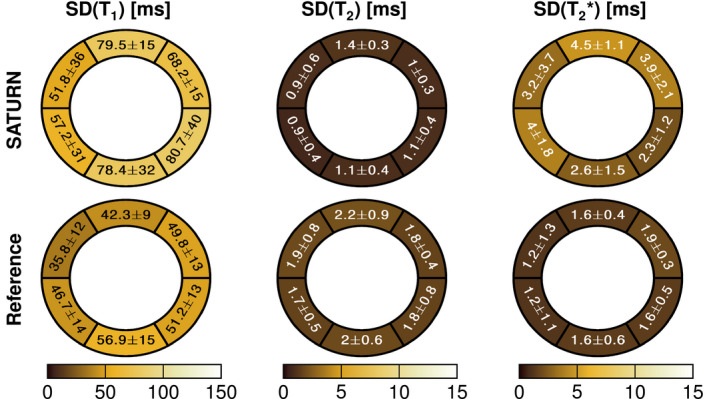
Bullseye plot of $T_{1}$, $T_{2}$, and $T_{2}^{*}$ relaxation times acquired with SATURN and the single‐parameter reference. The bullseye values are reported as the within‐segment mean ± the within‐segment standard deviation of the standard deviation map (SD map) for each segment. The voxel‐wise standard deviation was higher for $T_{1}$ and $T_{2}^{*}$ obtained with SATURN and smaller for $T_{2}$ compared with the single‐parameter methods

SD maps are calculated for all healthy subjects for SATURN and the conventional methods and resulted in mean values of $\sigma (T_{1})=68\;\text{ms}$, $\sigma (T_{2})=1.1\;\text{ms}$ and $\sigma (T_{2}^{*})=3.3\;\text{ms}$ and for the conventional methods $\sigma (T_{1})=39.3\;\text{ms}$, $\sigma (T_{2})=1.9\;\text{ms}$ and $\sigma (T_{2}^{*})=1.5\;\text{ms}$. Examples of SD maps are shown in Figure 5B and Supporting Information Figure S2. Figure 8 shows the mean and the SD of the calculated SD maps in each of the 6 segments. For $T_{1}$, SATURN achieved 23.3% lower within‐segment SDs and improved precision compared with SASHA $T_{1}$ map. $T_{2}$ shows comparable precision between SATURN and the single‐parameter method (5.1% deviations). Increased within‐segment SDs of 8.3% are observed for SATURN $T_{2}^{*}$ compared with the reference multi‐GRE.

**FIGURE 9:**
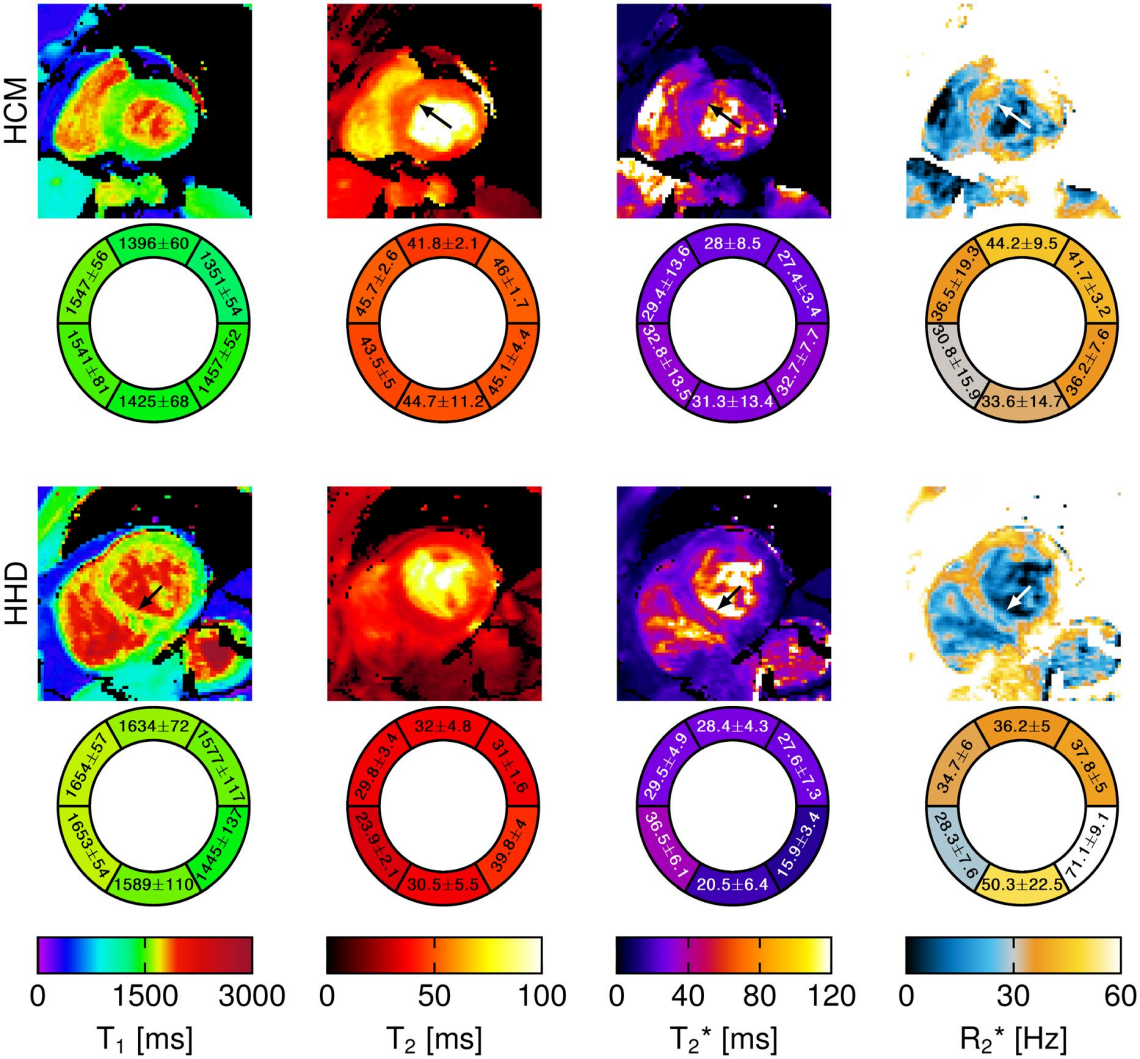
SATURN $T_{1}$, $T_{2}$, and $T_{2}^{*}$ maps for a patient with hypertrophic cardiomyopathy (HCM) and 1 patient with suspected hypertense heart disease (HHD). The corresponding bullseye plots are shown respectively. In the patient with HCM, increased $T_{1}$, $T_{2}$, and $T_{2}^{*}$ were observed in the septal region. For the patient with HHD, increased $T_{1}$ and $T_{2}^{*}$ were observed in the septal region as well as patchy structures in the $T_{1}$ map

Figure 9 shows SATURN $T_{1}$, $T_{2}$, and $T_{2}^{*}$ maps for a patient with HCM, and 1 patient with suspected HHD and the corresponding bullseye plots. Increased $T_{1}$, $T_{2}$, and $T_{2}^{*}$ times (1607/47.0/35.5 ms vs 1487/38.5/26.5 ms) are observed in the septal regions compared with the lateral myocardium in the patient with HCM. SATURN shows increased $T_{1}$ times and patchy structures in the patient with HHD. $T_{2}^{*}$ times are substantially elevated.

## 4 DISCUSSION

In this study, we proposed the SATURN sequence for free‐breathing simultaneous quantification of $T_{1}$, $T_{2}$, and $T_{2}^{*}$ in the myocardium based on a gradient‐echo readout in combination with saturation pulses and $T_{2}$ preparation pulses. We demonstrated good agreement with Bloch simulations and phantom experiments yielding generally accurate $T_{1}$ times. However some biases for $T_{2}$ and $T_{2}^{*}$ are observed. In vivo measurements provided robust image quality comparable to reference methods for all segments in the mid‐ventricular short‐axis view.

$T_{1}$ measurements resulted in good accuracy compared to spin‐echo sequences and SASHA in the phantom and in vivo. Mean $T_{1}$ times in the 6 segments are comparable to previously reported values for saturation based $T_{1}$ mapping at 3T.^12^, ^50^ $T_{1}$ maps yielded similar image quality and smaller within‐segment standard deviations compared with SASHA. Similar inter‐subject variability was found between SATURN and the reference method.

Our simulations indicate that accuracy in $T_{2}$ mapping is compromised for long $T_{1}$/$T_{2}$ combinations due to insufficient recovery during the rest‐period. However, as this effect is only marked at values outside the relevant in vivo range, a choice of 4 seconds rest periods seemed justified. In vivo $T_{2}$ measurements resulted in lower $T_{2}$ times than reported in literature,^22^, ^51^, ^52^ but only minor differences were observed between SATURN and the reference $T_{2}$‐prepared bSSFP. The lower $T_{2}$ times obtained in this study as compared to previous literature^22^, ^51^, ^52^ are related to the use of a three‐parameter fit model, which was previously shown to yield lower $T_{2}$ times (Supporting Information Figure S4). Other than that, we observed a drop of $T_{2}$ in mid‐inferior segments in some healthy subjects due to $B_{1}^{+}$ inhomogeneities, which could be corrected by better shimming routines. We used rest periods before the $T_{2}$ preparations instead of saturation pulses directly after the ECG trigger because the SNR of the $T_{2}$‐prepared images for the gradient‐echo readout was too low for accurate $T_{2}$ quantification as numerical simulations showed.^53^ $T_{2}$ maps in vivo and in phantom appeared visually smoother and more blurred as compared with the conventional single‐parameter maps due to the centric k‐space reordering in SATURN. With centric k‐space reordering, the magnetization transfer function acts as a slight low pass filter.^54^ We decided in favor of centric k‐space reordering due to the improved quantification result and image quality, especially for $T_{1}$.

Bloch simulations without noise result in accurate $T_{2}^{*}$ quantification. However, phantom measurements resulted in deviations of up to 20%, likely due to susceptibility artifacts and increased noise, as this was the dominant factor in the Bloch simulations. Especially for the tubes with very high $T_{2}^{*}$ times the quantification in the phantom failed, which might be due to the very short maximum TE of the 5 echoes from SATURN. However, for $T_{2}^{*}$ in the in vivo relevant range SATURN was still observed to be more accurate than the reference GRE method. $T_{2}^{*}$ times in vivo are in the range of reported literature^55^, ^56^, ^57^ and slightly increased compared with the reference GRE (5.9%). The overestimation is likely linked to a shorter maximum TE. However, increased $T_{2}^{*}$ times are measured without truncation due to sufficient SNR.^36^, ^57^ A drop in $T_{2}^{*}$ was observed in the mid‐inferior segment due to $B_{1}^{+}$ inhomogeneities as also observed for $T_{2}$.

Higher accelerations might be necessary for patients with high heart rates to reduce the time per single‐shot acquisition. The variability in $T_{1}$ maps is increased when using higher acceleration factors (GRAPPA *R* = 4). However, this can be alleviated by using regularization (SPIRiT + LLR) at the cost of inducing complexity in the post‐processing. $T_{2}$ maps reconstructed using acceleration factors of *R* = 3 and *R* = 4 resulted in visually similar $T_{2}$ maps with only slight deviations of 2.1% in the $T_{2}$ times and 1.9% in the within‐segment standard deviations. Similar to $T_{1}$, for the $T_{2}^{*}$ the use of *R* = 4 increases the within‐segment standard deviation by 52.4%, which might be due to the low SNR for images with long TEs.

In the patient with HCM we observed an increased $T_{1}$, $T_{2}$, and $T_{2}^{*}$ time as reported in literature. ^58^, ^59^, ^60^ Image quality was visually good. For the patient with suspected HHD, increased $T_{1}$ and $T_{2}^{*}$ was observed in the septal region and patchy structures in the $T_{1}$ map as typically observed in HHD.^61^ No reference methods were acquired in patients, which will be evaluated in future work.

Simultaneous measurements of $T_{1}$, $T_{2}$, and $T_{2}^{*}$ is more time‐efficient since all parameters are acquired in 1 scan (average acquisition time was 26.5±14.9seconds). Additionally, they share the same volume and are, therefore, inherently co‐registered. This eases the fusion of imaging information as corresponding regions are easy to identify. Furthermore, the assessment of multiple quantitative measures increases the specificity for diagnosis.^1^, ^2^, ^51^

Free‐breathing imaging was achieved by using a prospective navigator on the liver diaphragm. This may minimize the susceptibility to incomplete breath‐holds as often observed in patients suffering from dyspnea. Residual motion is compensated by the use of image registration. We used rigid‐registration as previously reported to yield satisfactory results in healthy subjects (Supporting Information Figure S5).^62^ In patients with variable breathing patterns and/or arrhythmia, the motion correction for respiratory as well as the cardiac cycle might be improved by using non‐rigid registrations, which is subject of future work. In addition, simultaneous multislice acquisition^63^ can be used to cover multiple slices per acquisition, which enables whole heart imaging in a relatively short time.

Intramyocardial fat is often present in cardiac patients and is known to shorten the $T_{1}$ and $T_{2}$ times.^64^ While variable impact of the fat fraction on bSSFP based cardiac relaxometry has been reported,^65^ the effects on GRE‐based mapping, as proposed in this study, are expected to be affected by fewer confounders. Furthermore, in the presence of substantial intramyocardial fat, the $T_{2}^{*}$ decay deviates from a monoexponential decay. Dixon‐encoding mapping might be used to separate the fat and water signal and overcome the deviations in the quantitative measures.^66^, ^67^ Integration of these techniques in our proposed sequence and dedicated evaluation for fatty storage disease warrant further investigation.

A physics‐based 5‐parameter model was used for the quantification. Recent trends emerged using machine learning for improving the reconstruction and fitting with non‐explicit modeling and might be applied due to the limited spatial resolution, partial volume effects, and noise.^68^, ^69^, ^70^, ^71^

This study has several limitations. Saturation recovery based methods for $T_{1}$ quantification suffer from a decreased dynamic range of the $T_{1}$ recovery curve, which is known to decrease the precision.^38^ However, compared with inversion recovery methods such as MOLLI, the accuracy is not impacted^72^ (Supporting Information Figure S1). The dynamical range could be increased by shifting the readout to the succeeding heart‐beat, as previously reported.^73^ However, in this case, navigator gating may affect the sampling of the saturation recovery. Nonetheless, this modification may lead to valuable improvements in terms of map quality for tachycardiac patients and warrants further investigation. Single‐shot imaging suffers from long readout blocks, especially for a multi‐gradient‐echo readout with 5 echoes. Higher heart rates will result in more cardiac motion during the acquisition. Therefore, the maximal TR of the echoes has to be short enough to acquire the whole k‐space in 1 diastolic phase. However, short TR reduces the accuracy of the $T_{2}^{*}$ quantification of long $T_{2}^{*}$ times as observed under certain circumstances or lower field‐strength. Higher acceleration factors enable the sampling of longer echo times in the same acquisition window, albeit at the cost of reduced SNR. We showed that this limitation might be partially compensated for by the use of regularization when using acceleration factors higher than *R* = 3. A maximum TE of 8.6ms is short compared with conventional methods that often use a maximum TE around 16‐18 ms.^20^ We decided to use a truncation fitting model to increase the quantification accuracy, especially for the low SNR contrasts 4 and 6.^36^ Nevertheless, the use of short echo times might lead to an overestimation of $T_{2}^{*}$. However, an increase of 1.5 ms in $T_{2}^{*}$ in vivo compared with the conventional multi‐GRE was obtained with SATURN. This deviation is explained by the shorter maximum TE relative to the reference method. Increasing the length of the GRE readout train may be considered in a trade‐off against higher acceleration rates if improved accuracy for long $T_{2}^{*}$ is desired. Faster acquisition schemes such as radial single‐shot images might offer a better compromise between longer TE and short enough acquisition windows, which will be evaluated in further research. Conventionally, $T_{2}^{*}$ maps are acquired with lower spatial resolution compared with $T_{1}$ and $T_{2}$. Since we are measuring all 3 parameters from the same scan with the same spatial resolution we acquire with slightly higher resolution for $T_{2}^{*}$ as commonly acquired.^20^ Furthermore, it is generally recommended to perform $T_{2}^{*}$ mapping at 1.5T. Hence, the quality of the $T_{2}^{*}$ quantification might show superior results at 1.5T. Blood signal suppression is also often used in $T_{2}^{*}$ mapping to alleviate partial volume effects. However, in this study, we refrained from additional blood signal suppression but may benefit from decreased partial voluming due to an increased imaging resolution.

## 5 CONCLUSION

SATURN enables joint quantification of the most relevant clinical relaxation times, $T_{1}$, $T_{2}$, and $T_{2}^{*}$, with robust image quality in a single free‐breathing scan. Good quantification accuracy was demonstrated in a phantom. In vivo free‐breathing imaging yielded high visual image quality.

## Supporting information


**TABLE S1** Phantom $T_{1}$, $T_{2}$, and $T_{2}^{*}$ times for SATURN, the conventional cardiac mapping sequences (SASHA, $T_{2}$‐prepared bSSFP, multi‐GRE) and the reference SE and GRE methods for all single tubes
**TABLE S2** $T_{1}$, $T_{2}$, and $T_{2}^{*}$ times for the in vivo measurements for SATURN compared with the conventional cardiac mapping sequences (SASHA, $T_{2}$‐prepared bSSFP, multi‐GRE) across all healthy subjects. Per‐subject relaxation times are summarized as means and within‐segment standard deviation, as highlighted in blue. The corresponding P‐values for the t‐test with Bonferroni correction are shown below
**FIGURE S1** Simulations for the proposed SATURN sequence for varying $T_{1}$ (left), $T_{2}$ (middle), and $T_{2}^{*}$ (right) for different sources of error as (A) the rest period before the $T_{2}$ preparations, (B) Rician noise on the signal with corresponding SNR, (C) the heart rate in beats‐per‐minute (bpm) and (D) the $T_{2}$ preparation efficiency as a scale factor of the flip down and flip up 90^∘^ pulses of the $T_{2}$ preparation module. The relative deviation between simulated and true quantitative measures is depicted for each source of error. All simulations are performed with the common parameters (rest period of 10 seconds, noise‐free, heart rate of 60 bpm, and $T_{2}$ preparation efficiency in %) and only the source of error was varied. In A, only deviations in $T_{2}$ are observed for a rest period of shorter than 5 seconds. In B, major deviations are observed for $T_{2}^{*}$ dependent on the Rician noise. $T_{2}$ is less impacted and $T_{1}$ only slightly. C, no effect in neither $T_{1}$, $T_{2}$, and $T_{2}^{*}$ was observed dependent on the heart rate. Deviations would be assumed for $T_{1}$ only if noise was added. In D, a strong drop in $T_{2}$ is observed for a decreased $T_{2}$ preparation efficiency
**FIGURE S2** A, In vivo $T_{1}$, $T_{2}$, and $T_{2}^{*}$ maps acquired with single‐parameter reference methods (left) and the proposed SATURN sequence (right) for 2 healthy subjects. Visually homogeneous mapping is achieved throughout the myocardium for $T_{1}$ and $T_{2}$, minor artifacts appear in $T_{2}^{*}$ maps. Image quality appears visually comparable to the reference methods. B, Below the standard deviation (SD) maps are shown for the 3 relaxation times and the same subjects for SATURN and the reference methods
**FIGURE S3** $T_{1}$, $T_{2}$, $T_{2}^{*}$, and $R_{2}^{*}$ maps are shown for the acquisition with acceleration factor R=3 (left), R=4 (middle) and for R=4 with additional regularization using SPIRiT + locally low rank (LLR) regularization (right). Quantitative measures with the standard deviation (shaded area) extracted from the SD maps along the myocardial wall are shown on the right side for R=3 (blue), R=4 (orange), and SPIRiT + LLR (yellow). Visual image quality is improved and precision is regained after the use of SPIRiT + LLR for R=4. The color bar and the y‐axis of the plot have the same ranges. The corresponding quantitative times for the pixel‐wise curve are windowed the same as the color bar left of the axis. The LLR algorithm takes around 200 seconds on a single core
**FIGURE S4** In vivo $T_{2}$ times acquired with the $T_{2}$‐prepared bSSFP using a 3‐parameter fit model and 4 dynamics and a 2‐parameter fit model without the fourth dynamic (saturation). A, On the left side the mean $T_{2}$ times per healthy subject are correlated between the 2‐parameter fit model and the 3‐parameter fit model. B, On the right side, the Bland‐Altman plot between 2 and 3 parameters is shown with a significant difference and an average bias of 5.85 ms increased $T_{2}$ when using the 2‐parameter model. C, The representative $T_{2}$ maps are depicted with the corresponding bullseye plots (D) showing the within‐segment mean and within‐segment standard deviation across all subjects
**FIGURE S5** A, Native $T_{1}$, $T_{2}$, and $T_{2}^{*}$ maps without (top) and with motion correction using rigid registration (bottom). B, Magnitude images which indicate the difference encoded in blue and red between 2 images and the corresponding registered images below. On the left side contrast number 5 (max $T_{S}^{\max}$) was motion corrupted as also seen in the resulting $T_{1}$ map above. In the center image contrast number 3 (second $T_{2}$ preparation) was corrupted and on the right image along the different gradient echoes small translation was corrected. C, Signal intensity for a region of interest in the septal myocardium across 3 repetitions of the SATURN sequence
