# Supplementary material for: Free‐breathing simultaneous T 1, T 2, and T 2 ∗ quantification in the myocardium
Source: Magn Reson Med. 2021 Mar 29;86(3):1226–40. doi: 10.1002/mrm.28753 (PMC8252099; doi:10.1002/mrm.28753)
Supplement: Supplementary file 1 — TABLE S1 Phantom T1, T2, and T2∗ times for SATURN, the conventional cardiac mapping sequences (SASHA, T2‐prepared bSSFP, multi‐GRE) and the reference SE and GRE methods for all single tubes TABLE S2 T1, T2, and T2∗ times for the in vivo measurements for SATURN compared with the conventional cardiac mapping sequences (SASHA, T2‐prepared bSSFP, multi‐GRE) across all healthy subjects. Per‐subject relaxation times are summarized as means and within‐segment standard deviation, as highlighted in blue. The corresponding P‐values for the t‐test with Bonferroni correction are shown below FIGURE S1 Simulations for the proposed SATURN sequence for varying T1 (left), T2 (middle), and T2∗ (right) for different sources of error as (A) the rest period before the T2 preparations, (B) Rician noise on the signal with corresponding SNR, (C) the heart rate in beats‐per‐minute (bpm) and (D) the T2 preparation efficiency as a scale factor of the flip down and flip up 90∘ pulses of the T2 preparation module. The relative deviation between simulated and true quantitative measures is depicted for each source of error. All simulations are performed with the common parameters (rest period of 10 seconds, noise‐free, heart rate of 60 bpm, and T2 preparation efficiency in %) and only the source of error was varied. In A, only deviations in T2 are observed for a rest period of shorter than 5 seconds. In B, major deviations are observed for T2∗ dependent on the Rician noise. T2 is less impacted and T1 only slightly. C, no effect in neither T1, T2, and T2∗ was observed dependent on the heart rate. Deviations would be assumed for T1 only if noise was added. In D, a strong drop in T2 is observed for a decreased T2 preparation efficiency FIGURE S2 A, In vivo T1, T2, and T2∗ maps acquired with single‐parameter reference methods (left) and the proposed SATURN sequence (right) for 2 healthy subjects. Visually homogeneous mapping is achieved throughout the myocardium for T1 and T2, minor artifacts appear [file MRM-86-1226-s001.pdf]

SUPPLEMENTARY MATERIAL

The supplementary material is for both review and online publication. It is listed in numerically below with corresponding captions.

Supplementary Tables

Supporting Information Table S 1: Phantom T_1, T_2, and T_2^* times for SATURN, the conventional cardiac mapping sequences (SASHA, T_2-prepared bSSFP, multi GRE) and the reference SE and GRE methods for all single tubes.

	T_1 **[ms]**		T_2 **[ms]**		T_2^* **[ms]**	
Tube	SATURN<br>SASHA	Ref. IR-SE	SATURN<br>T_2 bSSFP	Ref. SE	SATURN<br>Multi GRE	Ref. GRE
1	357.9 ±4.2<br>312.0 ±7.8	320.1 ± 11.6	45.2 ±0.6<br>41.6 ±0.9	45.5 ± 0.7	55.6 ±3.8<br>40.7 ±1.8	47.8 ± 2.1
2	619.6 ±2.8<br>583.3 ±6.4	597.8 ± 9.0	48.3 ±0.8<br>44.6 ±0.8	46.1 ± 0.9	51.4 ±2.0<br>46.3 ±1.3	56.3 ± 1.6
3	475.7 ±2.9<br>442.8 ±7.2	463.4 ± 5.5	46.2 ±0.6<br>44.1 ±1.0	45.1 ± 0.6	53.9 ±2.0<br>45.0 ±1.3	58.1 ± 2.2
4	1092.3 ±7.7<br>1085.3 ±17.7	1125.1 ± 8.8	50.2 ±0.4<br>48.3 ±1.4	49.6 ± 0.9	62.5 ±4.5<br>49.7 ±2.9	60.7 ± 4.1
5	1690.2 ±28.0<br>1663.7 ±39.1	1764.1 ± 22.0	49.1 ±0.7<br>48.7 ±0.7	50.6 ± 1.5	52.7 ±7.2<br>50.5 ±2.8	60.8 ± 1.8
6	1432.6 ±20.0<br>1384.8 ±33.5	1475.4 ± 28.7	49.4 ±1.1<br>48.9 ±1.6	49.6 ± 1.1	42.9 ±3.7<br>52.6 ±4.9	60.5 ± 2.6
7	2091.9 ±34.4<br>2129.7 ±68.2	2253.7 ± 56.5	126.7 ±2.4<br>149.4 ±4.2	166.9 ± 1.8	475.1 ±224.8<br>166.7 ±30.1	198.7 ± 35.4
8	2618.7 ±55.7<br>2712.8 ±106.8	2950.5 ± 150.0	83.6 ±0.6<br>94.5 ±1.3	103.1 ± 2.6	389.1 ±146.7<br>109.3 ±12.4	139.1 ± 13.5
9	851.5 ±41.8<br>754.8 ±12.0	793.4 ± 15.9	97.1 ±4.0<br>109.5 ±2.9	104.6 ± 4.3	140.5 ±44.8<br>115.6 ±21.2	187.5 ± 55.5

Supporting Information Table S 2: T_1, T_2, and T_2^* times for the in vivo measurements for SATURN compared with the conventional cardiac mapping sequences (SASHA, T_2-prepared bSSFP, multi GRE) across all healthy subjects. Per-subject relaxation times are summarized as means and within-segment standard deviation, as highlighted in blue. The corresponding p-values for the t-test with Bonferroni correction are shown below.

	T_1 **[ms]**		T_2 **[ms]**		T_2^* **[ms]**	
Subject	SATURN	SASHA	SATURN	T_2 bSSFP	SATURN	Multi GRE
1	1611 ± 122	1620 ± 144	37.5 ± 3.4	33.8 ± 3.4	25.5 ± 7.4	23.6 ± 4.7
2	1530 ± 82	1583 ± 121	23.7 ± 2.9	33.5 ± 3.5	29.4 ± 7.4	28.2 ± 9.9
3	1626 ± 62	1575 ± 110	34.0 ± 4.4	33.5 ± 3.6	25.4 ± 4.8	22.6 ± 4.0
4	1508 ± 66	1546 ± 101	33.2 ± 3.9	26.6 ± 4.0	24.0 ± 6.2	24.7 ± 4.6
5	1588 ± 75	1535 ± 141	36.8 ± 2.8	36.7 ± 3.5	25.8 ± 6.1	21.2 ± 6.1
6	1586 ± 100	1488 ± 121	28.9 ± 3.3	33.1 ± 3.2	22.5 ± 6.3	21.9 ± 5.3
7	1543 ± 110	1491 ± 93	40.8 ± 4.6	35.2 ± 4.3	28.1 ± 6.0	27.4 ± 6.4
8	1536 ± 80	1504 ± 56	36.3 ± 2.3	34.9 ± 3.5	23.3 ± 4.4	22.2 ± 5.3
9	1512 ± 45	1537 ± 81	26.9 ± 4.8	28.5 ± 3.5	30.9 ± 7.8	28.0 ± 5.7
10	1689 ± 115	1559 ± 105	34.0 ± 3.0	35.6 ± 3.2	18.4 ± 4.6	18.3 ± 3.5
Mean	1573±86	1544±107	33.2±3.6	33.2±3.6	25.3±6.1	23.8±5.5
T-test	$p = 0.22$		$p = 0.98$		$p = 0.33$	

p-values less than 0.05 were considered significant. No significant difference between SATURN and the reference single parameter methods was found.

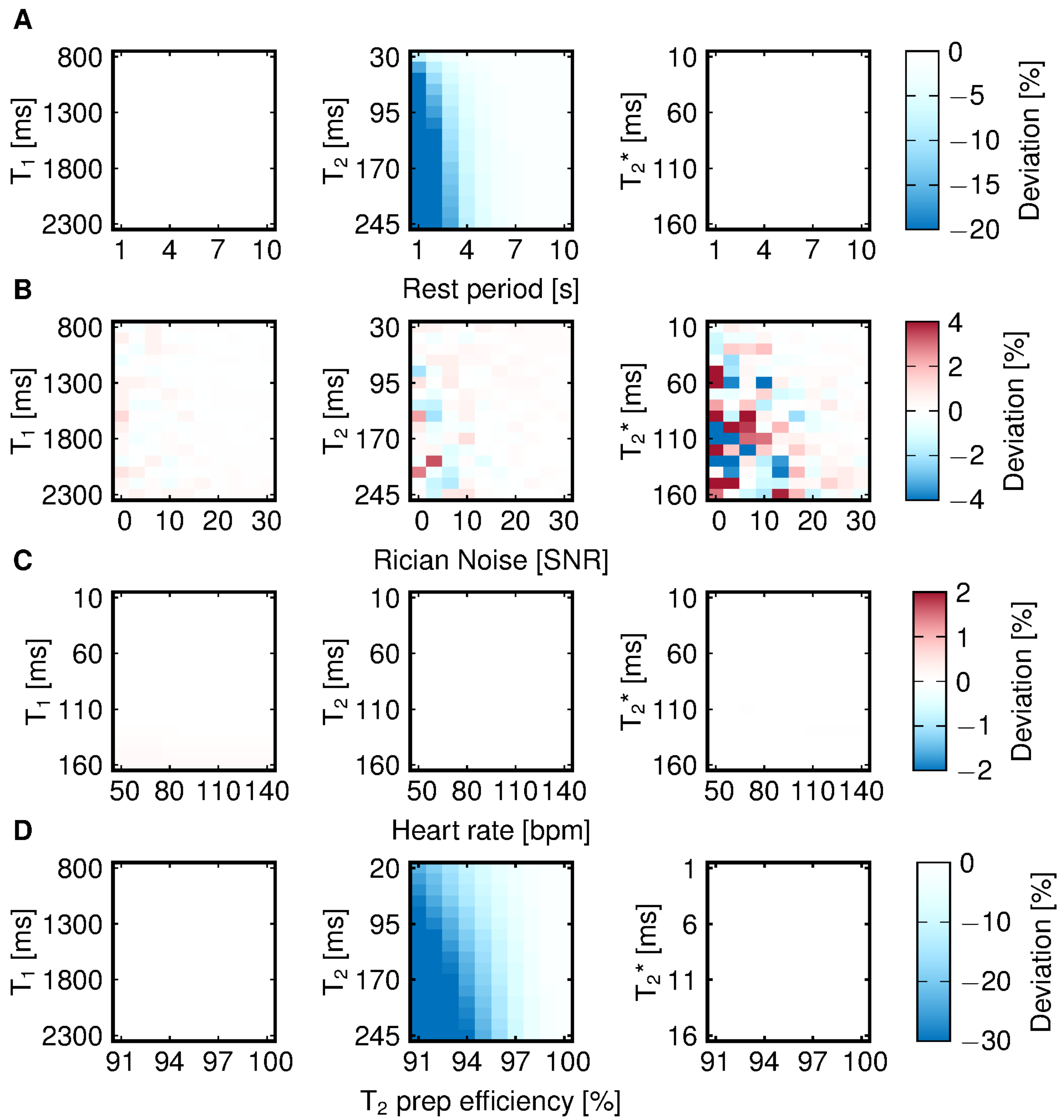

Supporting Information Figure S 1: Simulations for the proposed SATURN sequence for varying T_1 (left), T_2 (middle) and T_2^* (right) for different sources of error as (**A**) the rest period before the T_2 preparations, (**B**) Rician noise on the signal with corresponding SNR, (**C**) the heart rate in beats-per-minute (bpm) and (**D**) the T_2 preparation efficiency as a scale factor of the flip down and flip up 90° pulses of the T_2 preparation module. The relative deviation between simulated and true quantitative measures is depicted for each source of error. All simulations are performed with the common parameters (rest period of ten seconds, noise-free, heart rate of 60 bpm and T_2 preparation efficiency in %) and only the source of error was varied. In **A** only deviations in T_2 are observed for a rest period of shorter than 5 seconds. In **B** major deviations are observed for T_2^* dependent on the Rician noise. T_2 is less impacted and T_1 only slightly. **C** no effect in neither T_1, T_2, and T_2^* was observed dependent on the heart rate. Deviations would be assumed for T_1 only if noise was added. In **D** a strong drop in T_2 is observed for a decreased T_2 preparation efficiency.

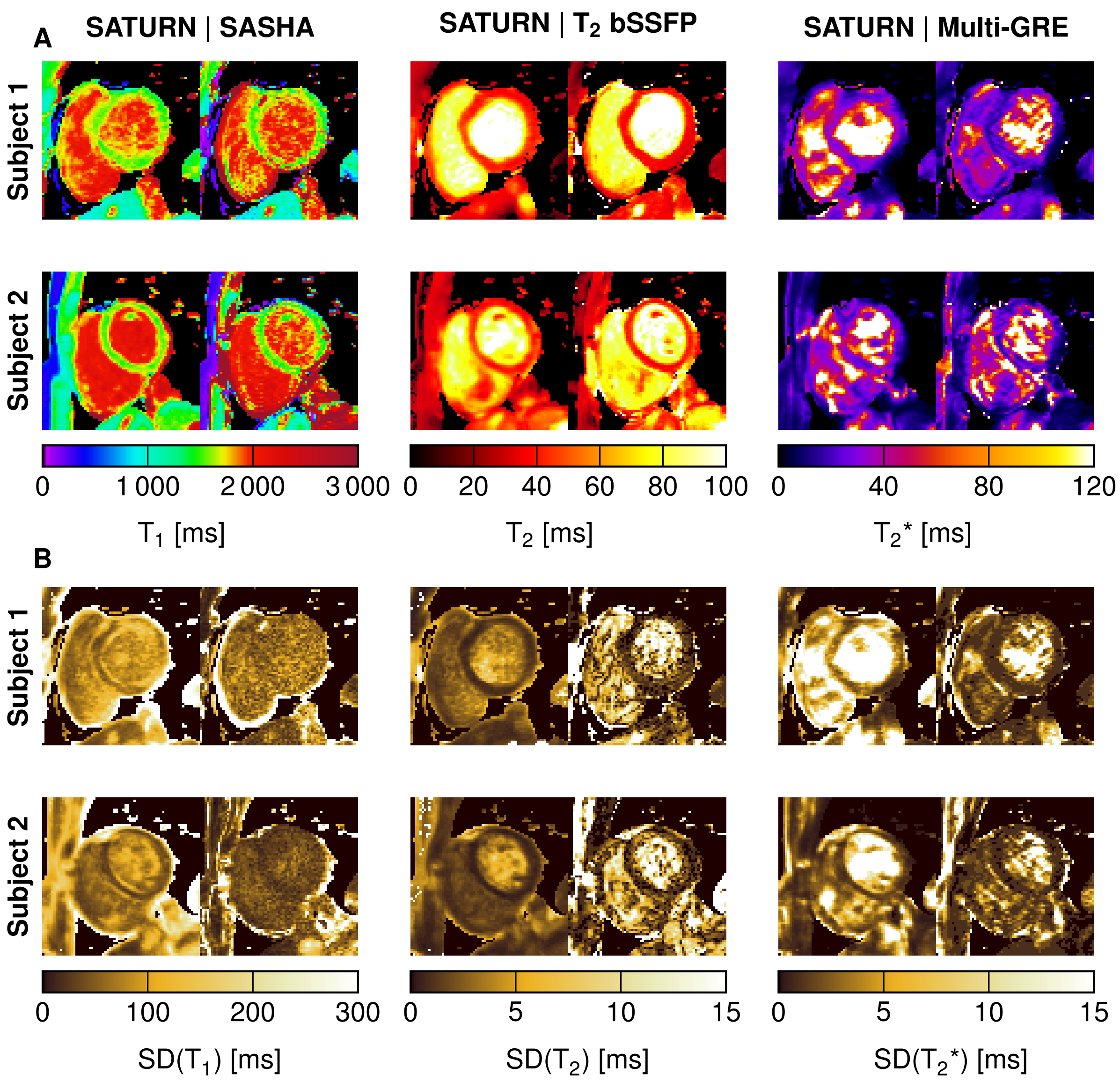

Supporting Information Figure S 2: A) In vivo T_1, T_2, and T_2^* maps acquired with single parameter reference methods (left) and the proposed SATURN sequence (right) for two healthy subjects. Visually homogeneous mapping is achieved throughout the myocardium for T_1 and T_2, minor artifacts appear in T_2^* maps. Image quality appears visually comparable to the reference methods. **B)** Below the standard deviation (SD) maps are shown for the three relaxation times and the same subjects for SATURN and the reference methods.

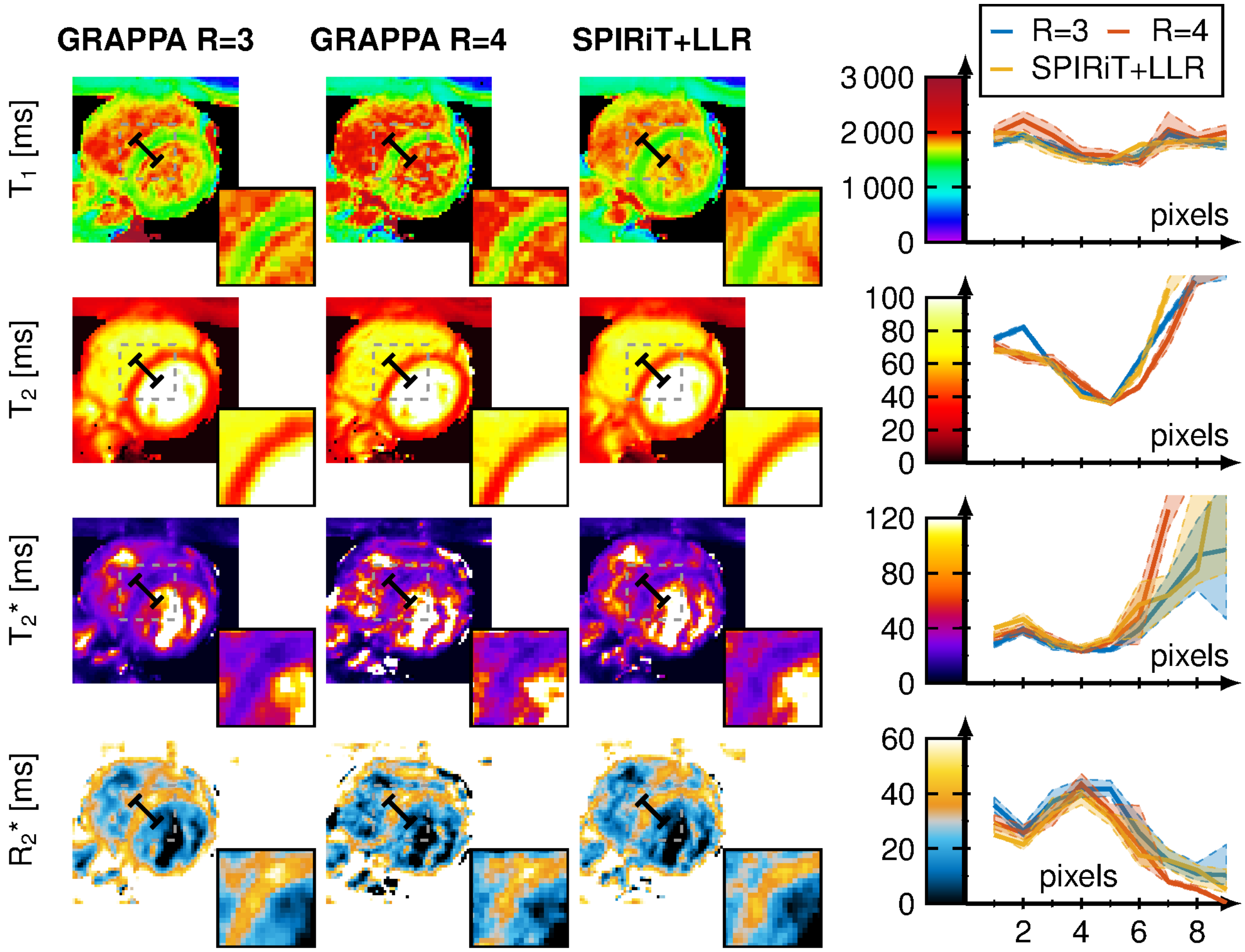

Supporting Information Figure S 3: T_1, T_2, T_2^*, and R_2^* maps are shown for the acquisition with acceleration factor R=3 (left), R=4 (middle) and for R=4 with additional regularization using SPIRiT + locally-low rank (LLR) regularization (right). Quantitative measures with the standard deviation (shaded area) extracted from the SD maps along the myocardial wall are shown on the right side for R=3 (blue), R=4 (orange), and SPIRiT + LLR (yellow). Visual image quality is improved and precision is regained after the use of SPIRiT + LLR for R=4. The color bar and the y-axis of the plot have the same ranges. The corresponding quantitative times for the pixel-wise curve are windowed the same as the color bar left of the axis. The LLR algorithm takes around 200 seconds on a single core.

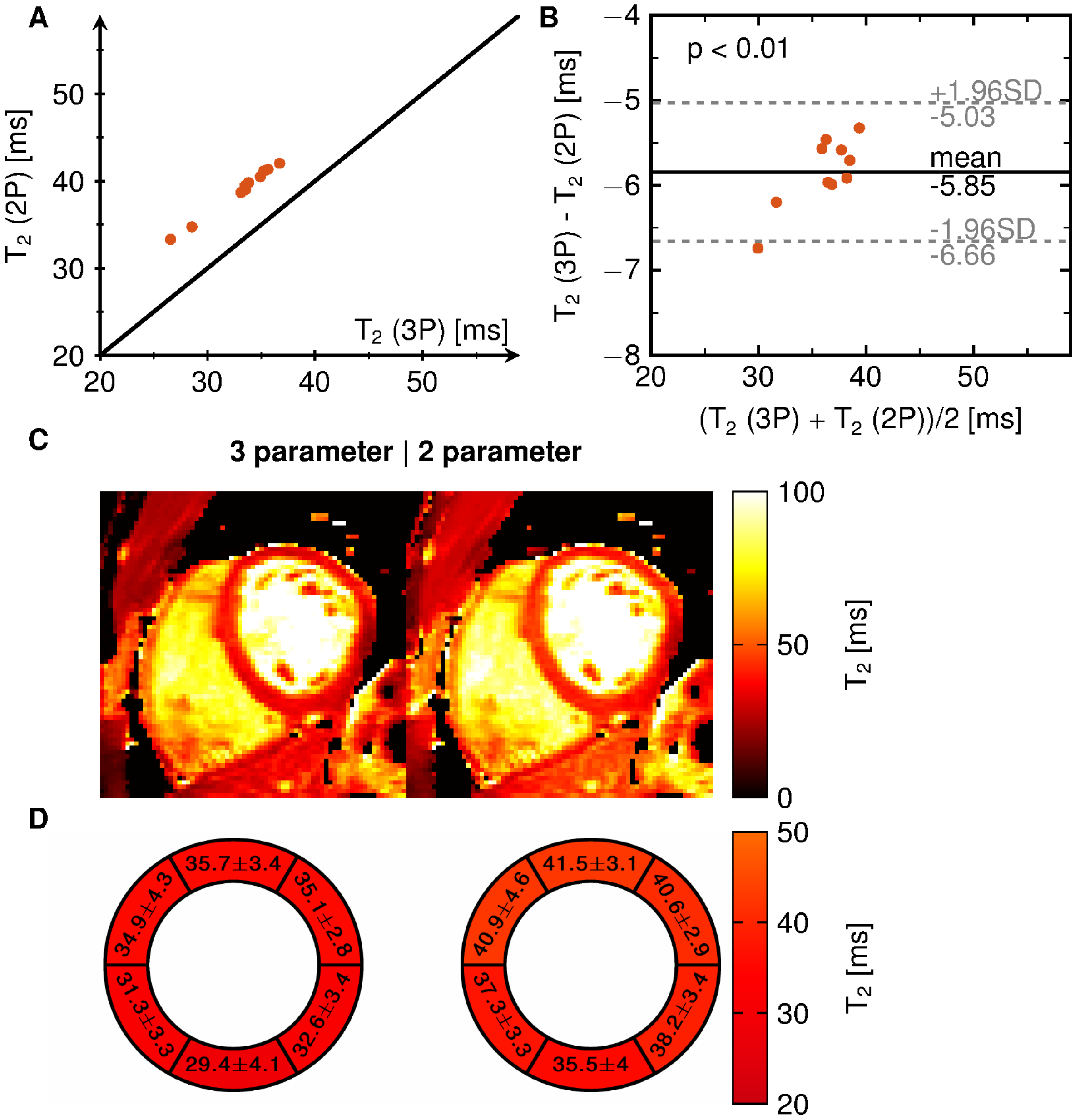

Supporting Information Figure S 4: In vivo T_2 times acquired with the T_2-prepared bSSFP using a 3 parameter fit model and 4 dynamics and a 2 parameter fit model without the fourth dynamic (saturation). **A)** On the left side the mean T_2 times per healthy subject are correlated between the 2 parameter fit model and the 3 parameter fit model. **B)** On the right side the Bland Altman plot between 2 and 3 parameters is shown with a significant difference and an average bias of 5.85 ms increased T_2 when using the 2 parameter model. **C)** The representative T_2 maps are depicted with the corresponding bullseye plots (**D**) showing the within-segment mean and within-segment standard deviation across all subjects.

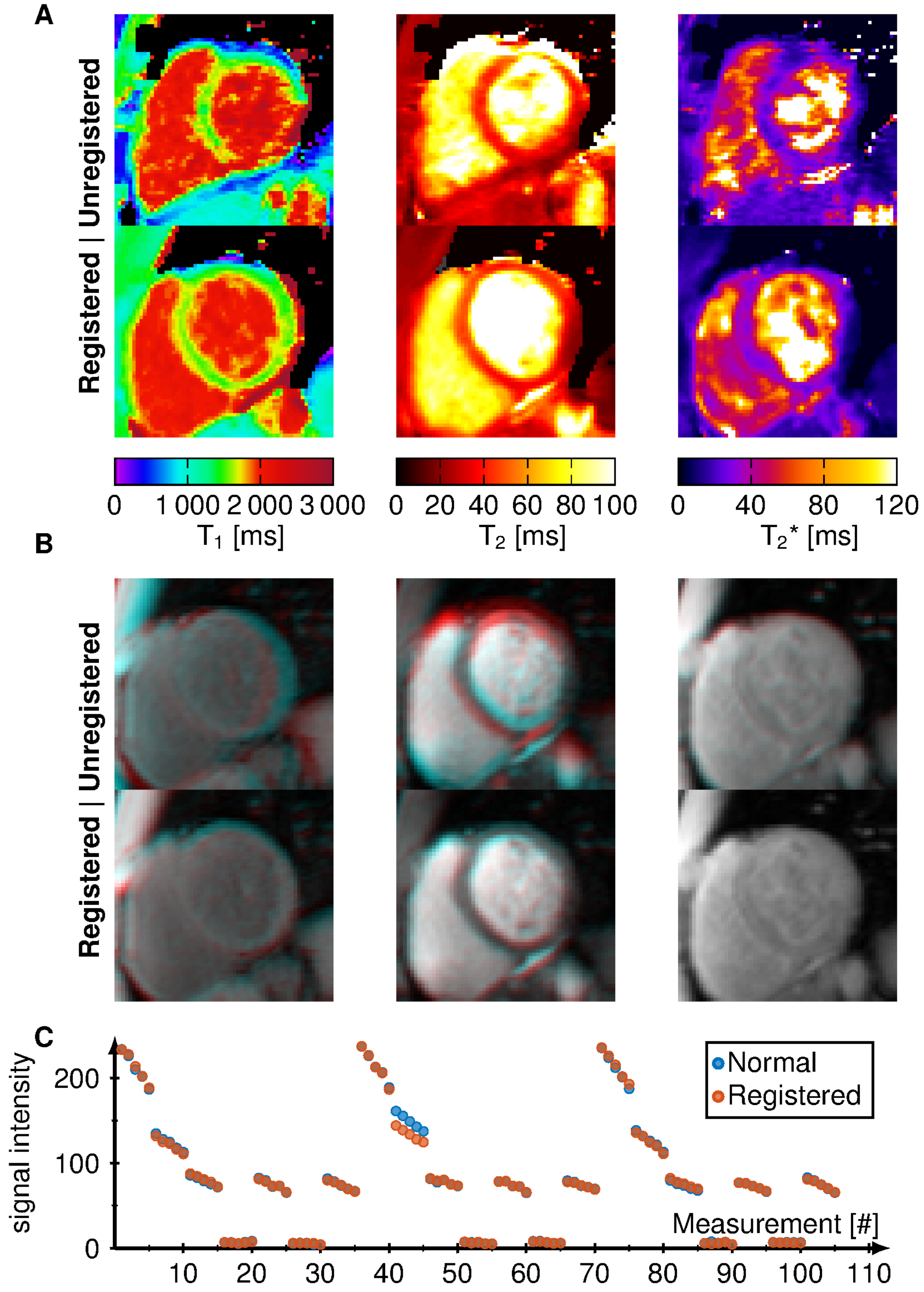

Supporting Information Figure S 5: A) Native T_1, T_2, and T_2^* maps without (top) and with motion correction using rigid registration (bottom). **B)** Magnitude images which indicate the difference encoded in blue and red between two images and the corresponding registered images below. On the left side contrast number five (max T_S^{max}) was motion corrupted as also seen in the resulting T_1 map above. In the center image contrast number three (second T_2 preparation) was corrupted and on the right image along the different gradient echoes small translation was corrected. **C)** Signal intensity for a region of interest in the septal myocardium across 3 repetitions of the SATURN sequence.
